# Supplementary material for: Flax latitudinal adaptation at LuTFL1 altered architecture and promoted fiber production
Source: Sci Rep. 2019 Jan 30;9:976. doi: 10.1038/s41598-018-37086-5 (PMC6354013; doi:10.1038/s41598-018-37086-5)
Supplement: Supplementary file 1 — Supplementary Information [file 41598_2018_37086_MOESM1_ESM.pdf]

Flax latitudinal adaptation at *LuTFL1* altered architecture and promoted fiber production.

Rafal M Gutaker[1,2], Maricris Zaidem[2], Yong-Bi Fu[3], Axel Diederichsen[3], Oliver Smith[1,4] , Roselyn Ware[1], Robin G Allaby[1]*

1. School of Life Sciences, University of Warwick, United Kingdom CV4 7AL
2. Max Planck Institute for Developmental Biology, Tübingen, Germany
3. Plant Gene Resources of Canada, Saskatoon Research and Development Centre, SK S7N 0X2, Canada
4. Centre for Geogenetics, Copenhagen University of Copenhagen, 1307 Copenhagen K, Denmark

Corresponding author r.g.allaby@warwick.ac.uk

Supplementary Tables

Table S1: list of putative flowering time gene homologs identified in the flax genome.

Locus	GBrowse CDS reference	E-value query *A. thaliana*	Specific primer sequence 5’ to 3’	Genomic position
LuTFL1	Lus10004886	9e-74	GGAGAAATCGATGGGGAAAG<br>TGGGTCGGTACTAACGCCT	scaffold1821:<br>167638..169735
LuTFL2	Lus10043385	4e-65	AATCGTAAAATTAACGGCGTC<br>AACCTCGACTATGTCGTCAGA	scaffold25:<br>2627196..2628849
LuTFL3	Lus10020600	2e-76	TGGGGCAAAATGGGGAAA<br>ATCGATCAATCTAGCGCCG	scaffold77:<br>545496..548022
LuTFL4	Lus10004884	1e-34	ATTAGGCAAAATGGGGAAAG<br>ATCGATCAGTTTAGCGCCG	scaffold1821:<br>157633..160714
LuTFL5	Lus10027442	9e-30	AGTTACAACAATGGCAAGAGG<br>TATTATTAGCGTTTTCTGGCG	scaffold96:<br>366083..367848
LuTFL6	Lus10005753	2e-26	AGTTACAAAAATGGCAAGAGGA<br>TGGCGGCATATTAGCGTT	scaffold1036:<br>75860..77589
LuTFL7	Lus10021372	2e-64	AAAAGATGGCAGCAGGTGC<br>TAGATCGATGTCATCGACGC	scaffold1123:<br>745123..747438
LuTFL8 (LuFT)	Lus10004452	2e-50	GTTATCGAAAATGCCAAGGG<br>GAGGATATCATCATCGCCGT	scaffold845:<br>154960..158446

Table S2: Pale and Cultivated flaxes used in this study.

Sample	Accession name	Donor	Donor accession	Country of origin	Collection date	Latitude	Longitiude	Improvement status	Cultivation purpose	Latitude data	LuTFL1	LuTFL 2	RADseq
Pale flaxes													
	W042	PGRC	CN113618	Turkey	2007	37.567	27.467	wild	NA	GPS	I	I	Included
	W043	PGRC	CN113630	Turkey	2007	41.183	33.733	wild	NA	GPS	III	I	Included
	W044	PGRC	CN107295	Greece	2000	39	22	wild	NA	Centroid	II	I	NA
	W045	PGRC	CN107296	Greece	2000	39	22	wild	NA	Centroid	II	I	Included
	W046	PGRC	CN19021	France	1998	46	2	wild	NA	Centroid	VIII	III	NA
	W047	PGRC	CN107257	France	1998	46	2	wild	NA	Centroid	VIII	III	Included
	W048	PGRC	CN113603	Turkey	2007	41.35	36.167	wild	NA	GPS	I	X	Included
	W049	PGRC	CN113606	Turkey	2007	41.383	36.183	wild	NA	GPS	I	I	Included
	W050	PGRC	CN113610	Turkey	2007	37.817	29.65	wild	NA	GPS	I	III	Included
	W051	PGRC	CN113621	Turkey	2007	37.033	27.367	wild	NA	GPS	I	I	Included
	W052	PGRC	CN113626	Turkey	2007	41.583	35.333	wild	NA	GPS	X	III	Included
	W053	PGRC	CN113629	Turkey	2007	41.217	33.233	wild	NA	GPS	III	I	Included
	W054	PGRC	CN113633	Turkey	2007	41.833	31.817	wild	NA	GPS	III	?	Included
	W055	PGRC	CN113636	Turkey	2007	40.433	29.933	wild	NA	GPS	III	I	Included
	W056	PGRC	CN113639	Turkey	2007	40.817	26.633	wild	NA	GPS	IX	I	Included
	W057	PGRC	CN113642	Turkey	2007	40.75	39.55	wild	NA	GPS	I	XI	Included
	W065	Coll.	W065	Croatia	2011	43.9	16.45	wild	NA	GPS	VIII	III	Included
	W066	Coll.	W066	Croatia	2011	43.467	16.833	wild	NA	GPS	III	III	Included
	W067	Coll.	W067	Croatia	2011	43.017	17.45	wild	NA	GPS	III	I	Included
	W068	Coll.	W068	Montenegro	2011	42.233	18.883	wild	NA	GPS	IV	I	Included
	W069	Coll.	W069	Albania	2011	41.167	19.467	wild	NA	GPS	III	I	Included
	W070	Coll.	W070	Greece	2011	39.9	20.367	wild	NA	GPS	VI	II	Included
	W072	Coll.	W072	Greece	2011	38.983	21.15	wild	NA	GPS	V	II	Included
	W074	Coll.	W074	Greece	2011	39.05	21.867	wild	NA	GPS	NA	I	NA
	W076	Coll.	W076	Greece	2011	39.783	21.633	wild	NA	GPS	II	I	Included
	W077	Coll.	W077	Greece	2011	39.967	21.5	wild	NA	GPS	II	I	Included
	W081	Coll.	W081	Greece	2011	40.35	23.933	wild	NA	GPS	III	I	Included
	W082	Coll.	W082	Greece	2011	40.583	23.783	wild	NA	GPS	III	I	Included
	W085	Coll.	W085	Bulgaria	2011	41.583	23.717	wild	NA	GPS	VII	III	Included
	W086	Coll.	W086	Bulgaria	2011	NA	NA	wild	NA	GPS	NA	NA	NA
	W094	Coll.	W094	Croatia	2011	45.13	18.233	wild	NA	GPS	VIII	III	Included
	W095	Coll.	W095	Croatia	2011	45.367	16.267	wild	NA	GPS	VIII	III	Included
	W096	Coll.	W096	Croatia	2011	45.417	15.367	wild	NA	GPS	VIII	III	NA
Cultivated flaxes													
	M056	PGRC	CN97321	Romania	1977	46§	25§	Cultivar	Oil	Centroid	NA	VI	Included
	M057	PGRC	CN97322	Romania	1977	46	25	Cultivar	Oil	Centroid	NA	VI	NA
	M068	PGRC	CN97044	Spain	1977	40	4	Cultivar	Oil	Centroid	NA	VI	NA
	M021	PGRC	CN18990	Czech Republic	1999	49.5	15.5	Cultivar	Fiber	Centroid	NA	X	NA
	M036	PGRC	CN19011	Tunisia	1999	34	9	Cultivar	Fiber	Centroid	NA	X	NA
	M010	PGRC	CN98561	Turkey	1977	39	35	Cultivar	Fiber	Centroid	I	NA	Included
	M043	PGRC	CN113611	Turkey	2007	39	35	Landrace	Unknown	Centroid	I	NA	Included
	M051	PGRC	CN98164	Iran	1977	32	53	Cultivar	Oil	Centroid	I	NA	Included
	M042	PGRC	CN100893	Iran	1999	32	53	Landrace	Unknown	Centroid	I	III	NA
	M053	PGRC	CN97017	Turkey	1977	39	35	Cultivar	Oil	Centroid	I	III	NA
	M059	PGRC	CN98278	Hungary	1977	47	20	Cultivar	Oil	Centroid	I	IV	NA
	M011	PGRC	CN98562	Turkey	1977	39	35	Cultivar	Fiber	Centroid	I	V	Included
	M001	PGRC	CN98833	Russia	1977	60	100	Dehiscent	Unknown	Centroid	I	VI	Included
	M003	PGRC	CN100852	Portugal	2002	39.5	8	Dehiscent	Unknown	Centroid	I	VI	Included
	M004	PGRC	CN97606	Spain	1977	40	4	Dehiscent	Unknown	Centroid	I	VI	Included
	M005	PGRC	CN98507	Israel	1977	31.5	34.75	Cultivar	Fiber	Centroid	I	VI	Included
	M007	PGRC	CN97048	Iran	1977	32	53	Cultivar	Fiber	Centroid	I	VI	NA
	M012	PGRC	CN96889	Turkey	1977	39	35	Cultivar	Fiber	Centroid	I	VI	NA
	M013	PGRC	CN98299	Hungary	1977	47	20	Cultivar	Fiber	Centroid	I	VI	NA
	M015	PGRC	CN98303	Hungary	1977	47	20	Cultivar	Fiber	Centroid	I	VI	NA
	M020	PGRC	CN97326	Poland	1977	52	20	Cultivar	Fiber	Centroid	I	VI	NA
	M044	PGRC	CN113613	Turkey	2007	39	35	Landrace	Unknown	Centroid	I	VI	Included
	M063	PGRC	CN97176	Czech Repbulic	1977	49.5	15.5	Cultivar	Oil	Centroid	I	VI	Included
	M067	PGRC	CN98552	UK	1977	54	2	Cultivar	Oil	Centroid	I	VI	NA
	M037	PGRC	CN100960	Tunisia	1999	34	9	Cultivar	Fiber	Centroid	I	IX	NA
	M050	PGRC	CN98163	Iran	1977	32	53	Cultivar	Oil	Centroid	I	IX	Included
	M025	PGRC	CN32544	Russia	1978	60	100	Cultivar	Fiber	Centroid	I	X	Included
	M049	PGRC	CN101028	Tunisia	1999	34	9	Landrace	Unknown	Centroid	I	X	NA
	M029	PGRC	CN101020	Kazakhstan	1999	48	68	Cultivar	Fiber	Centroid	III	NA	NA
	M071	PGRC	CN98880	Morocco	1977	32	5	Cultivar	Oil	Centroid	III	NA	NA
	M017	PGRC	CN18978	Germany	1990	51	9	Cultivar	Fiber	Centroid	III	III	NA
	M066	PGRC	CN98752	France	1977	46	2	Cultivar	Oil	Centroid	III	III	Included
	M016	PGRC	CN98475	Germany	1977	51	9	Cultivar	Fiber	Centroid	III	VI	NA
	M019	PGRC	CN97325	Poland	1977	52	20	Cultivar	Fiber	Centroid	III	VI	NA
	M023	PGRC	CN98479	Czech Republic	1977	49.5	15.5	Cultivar	Fiber	Centroid	III	VI	NA
	M035	PGRC	CN101025	Morocco	1999	32	5	Cultivar	Fiber	Centroid	III	VII	NA
	M052	PGRC	CN113643	Turkey	2009	39	35	Cultivar	Oil	Centroid	III	IX	Included
	M014	PGRC	CN98300	Hungary	1977	47	20	Cultivar	Fiber	Centroid	III	X	NA
	M018	PGRC	CN18991	Poland	1999	52	20	Cultivar	Fiber	Centroid	III	X	NA
	M024	PGRC	CN18995	Russia	1999	60	100	Cultivar	Fiber	Centroid	III	X	NA
	M026	PGRC	CN101099	Russia	1999	60	100	Cultivar	Fiber	Centroid	III	X	Included
	M027	PGRC	CN101056	Ukraine	1999	49	32	Cultivar	Fiber	Centroid	III	X	NA
	M030	PGRC	CN18988	France	1999	46	2	Cultivar	Fiber	Centroid	III	X	Included

Sample	Accession name	Donor	Donor accession	Country of origin	Collection date	Latitude	Longitiude	Improvement status	Cultivation purpose	Latitude data	LuTFL1	LuTFL 2	RADseq
	M030	PGRC	CN18988	France	1999	46	2	Cultivar	Fiber	Centroid	III	X	Included
	M031	PGRC	CN18982	France	1999	46	2	Cultivar	Fiber	Centroid	III	X	Included
	M038	PGRC	CN18999	The Netherlands	1999	52.5	5.75	Cultivar	Fiber	Centroid	III	X	NA
	M039	PGRC	CN100930	The Netherlands	1999	52.5	5.75	Cultivar	Fiber	Centroid	III	X	Included
	M040	PGRC	CN100929	The Netherlands	1999	52.5	5.75	Cultivar	Fiber	Centroid	III	X	Included
	M041	PGRC	CN100909	Palestine	1999	31.5	34.75	Landrace	Unknown	Centroid	III	X	NA
	M045	PGRC	CN101035	Russia	1999	60	100	Landrace	Unknown	Centroid	III	X	Included
	M046	PGRC	CN101046	Russia	1999	60	100	Landrace	Unknown	Centroid	III	X	Included
	M047	PGRC	CN100922	Malta	1999	35.5	14.583	Landrace	Unknown	Centroid	III	X	NA
	M060	PGRC	CN97758	Germany	1977	51	9	Cultivar	Oil	Centroid	III	X	NA
	M061	PGRC	CN97760	Germany	1977	51	9	Cultivar	Oil	Centroid	III	X	Included
	M062	PGRC	CN97332	Poland	1977	52	20	Cultivar	Oil	Centroid	III	X	Included
	M064	PGRC	CN97344	Czech Republic	1977	49.5	15.5	Cultivar	Oil	Centroid	III	X	NA
	M069	PGRC	CN98826	Egypt	1977	27	30	Cultivar	Oil	Centroid	III	X	Included
	M070	PGRC	CN97439	Egypt	1977	27	30	Cultivar	Oil	Centroid	III	X	Included
	M073	PGRC	CN30841	Kazakhstan	1976	48	68	Wild	NA	Centroid	VI	X	NA
	M065	PGRC	CN18996	France	1999	46	2	Cultivar	Oil	Centroid	VIII	X	Included
	M076	PGRC	CN30842	Kazakhstan	1976	48	68	Wild	NA	Centroid	XI	VI	Included
	M022	PGRC	CN98700	Czech Republic	1977	49.5	15.5	Cultivar	Fiber	Centroid	XII	NA	NA
	M033	PGRC	CN97043	Spain	1977	40	4	Cultivar	Fiber	Centroid	XII	VIII	NA
	M006	PGRC	CN98511	Israel	1977	31.5	34.75	Cultivar	Fiber	Centroid	XII	VI	Included
	M034	PGRC	CN101031	Morocco	1999	32	5	Cultivar	Fiber	Centroid	XII	VI	NA
	M048	PGRC	CN100896	Egypt	1999	27	30	Landrace	Unknown	Centroid	XII	VI	NA
	M054	PGRC	CN98987	Italy	1977	43	12.833	Cultivar	Oil	Centroid	XII	VI	NA
	M055	PGRC	CN98988	Italy	1977	43	12.833	Cultivar	Oil	Centroid	XII	VI	Included
	M058	PGRC	CN18992	Hungary	1999	47	20	Cultivar	Oil	Centroid	XII	VI	NA
	M028	PGRC	CN30862	Ukraine	1976	49	32	Cultivar	Fiber	Centroid	XII	X	NA
	M032	PGRC	CN18989	France	1999	46	2	Cultivar	Fiber	Centroid	XII	X	NA
	M074	PGRC	CN30844	Ukraine	1976	49	32	Wild	NA	Centroid	XII	X	Included
	M075	PGRC	CN30846	Ukraine	1976	49	32	Wild	NA	Centroid	XII	X	Included
	M072	PGRC	CN30852	Russia	1976	60	100	Wild	NA	Centroid	XIII	V	NA
	M009	PGRC	CN98162	Iran	1977	32	53	Cultivar	Fiber	Centroid	XIV	NA	Included
	M008	PGRC	CN97180	Iran	1977	32	53	Cultivar	Fiber	Centroid	XV	VI	Included
Historic flaxes													
	H095	VIR	VIR1727	Turkey	1926	38.833	35.633	landrace	Oil	GPS			Included
	H100	VIR	VIR2458	Turkey	1927	39.9	41.283	landrace	Oil	GPS			Included
	H098	VIR	VIR1931	Tunesia	1927	34	9	landrace	Intermediate	Centroid			Included
	H102	VIR	VIR3688	Latvia	1930	57.15	22.517	landrace	Fibre	GPS			Included
	H101	VIR	VIR2526	Italy	1927	39.217	9.1	landrace	Intermediate	GPS			Included
	H107	VIR	VIR1114	Israel	1923	32.05	34.75	cultivar	Intermediate	GPS			Included
	H086	VIR	VIR900	Italy	1923	43	12.833	landrace	Intermediate	GPS			Included
	H083	VIR	VIR776	Belarus	1923	55.167	30	landrace	Fibre	Centroid			Included
	H089	VIR	VIR929	Kazakhstan	1923	40.8	68.35	landrace	Oil	GPS			Included
	H106	VIR	VIR462	Turkey	1922	39	35	cultivar	Oil	Centroid			Included
	H088	VIR	VIR925	Armenia	1923	40.75	44.867	landrace	Oil	GPS			Included
	H093	VIR	VIR1693	Turkey	1926	38.5	27.5	landrace	Oil	GPS			Included
	H108	VIR	VIR3683	Latvia	1930	57.15	22.517	cultivar	Fibre	GPS			Included
	H112	VIR	VIR5546	Yugoslavia	1948	45.333	17.683	cultivar	Intermediate	GPS			Included
	H084	VIR	VIR791	Belarus	1923	53.017	30.65	landrace	Fibre	GPS			Included
	H085	VIR	VIR889	Ukraine	1923	49.717	28.85	landrace	Intermediate	GPS			Included
	H087	VIR	VIR901	Ukraine	1923	48.667	38.833	landrace	Intermediate	GPS			Included
	H090	VIR	VIR1028	Russia	1924	57.35	28.317	landrace	Fibre	GPS			Included
	H091	VIR	VIR1032	Russia	1924	57.017	29.333	landrace	Fibre	GPS			Included
	H092	VIR	VIR1042	Belarus	1924	55.183	30.183	landrace	Fibre	GPS			Included
	H094	VIR	VIR1723	Turkey	1926	38.833	35.633	landrace	Oil	GPS			Included
	H096	VIR	VIR1859	Iran	1926	35.25	60.617	landrace	Oil	GPS			Included
	H103	VIR	VIR17	Russia	1922	60	100	cultivar	Fibre	Centroid			Included
	H104	VIR	VIR193	The Netherlands	1922	52.5	5.75	cultivar	Fibre	Centroid			Included
	H105	VIR	VIR253	The Netherlands	1922	52.5	5.75	cultivar	Fibre	Centroid			Included
	H109	VIR	VIR3933	Germany	1930	51	9	cultivar	Fibre	Centroid			Included
	H110	VIR	VIR4165	France	1932	46	2	cultivar	Intermediate	Centroid			Included
	H111	VIR	VIR5339	Ukraine	1938	51.683	33.683	cultivar	Intermediate	GPS			Included
Herbaria flaxes													
	H031	HUO	HUO07	Uk	1875	54	2	Unknown	Unknown	Centroid			NA
	H050	HUNG	HUNG33	Turkey	1957	37	35.333	Unknown	Unknown	approximate GPS			NA
	H042	HUNG	HUNG07	Macedonia	1918	42	21.75	Unknown	Unknown	approximate GPS			NA
	H051	HUNG	HUNG34	Syria	1945	35	38.75	Unknown	Unknown	approximate GPS			NA
	H047	HUNG	HUNG12	Germany	1950	49.267	10.49	Unknown	Unknown	approximate GPS			NA
	H002	HPW	HPW03	Poland	1968	52.167	20.8	Unknown	Unknown	approximate GPS			NA
	H009	HPW	HPW13	Poland	1821	52	20	Unknown	Unknown	approximate GPS			NA
	H013	HPW	HPW17	Poland	1961	52	21	Landrace	Unknown	approximate GPS			NA
	H048	HUNG	HUNG13	France	1872	47.133	3.9	Unknown	Unknown	approximate GPS			NA
	H012	HPW	HPW16	Poland	1963	52.2	20.717	Landrace	Unknown	approximate GPS			NA

Table S3: Haplotype frequencies in wild and cultivated flax

Haplotype		Wild frequency	Cultivated frequency
LuTFL1			
	I	0.1935	0.2913
	II	0.129	0
	III	0.2903	0.5146
	IV	0.0323	0
	V	0.0323	0
	VI	0.0323	0.0097
	VII	0.0323	0
	VIII	0.1935	0.0097
	IX	0.0323	0.0097
	X	0.0323	0
	XI	0	0.0097
	XII	0	0.1165
	XIII	0	0.0097
	XIV	0	0.0097
	XV	0	0.0097
	XVI	0	0.0097
LuTFL2			
	I	0.5484	0.0202
	II	0.0645	0
	III	0.3226	0.0505
	IV	0	0.0101
	V	0	0.0505
	VI	0	0.3030
	VII	0	0.0101
	VIII	0	0.0101
	IX	0	0.0303
	X	0.0323	0.5151
	XI	0.0323	0

Table S4: Linkage Disequilibrium between the *LuTFL1* and *LuTFL2* loci

LuTFL1/LuTFL2	Wild				Cultivated			
	expected	observed	D	r	expected	observed	D	r
I/I	0.1061	0.0968	-0.0067	-0.0339	0.0059	0.0109	0.0046	0.0721
I/III	0.0624	0.0333	-0.0322	-0.1788	0.0147	0.0326	0.0172	0.1734
I/IV	-	-	-	-	0.0029	0.0108	0.0078	0.1716
I/VI	-	-	-	-	0.0883	0.1304	0.0475	0.2275
I/IX	-	-	-	-	0.0088	0.0217	0.0125	0.1608
I/X	0.0062	0.0333	0.0267	0.382	0.15	0.0435	-0.104	-0.4579
I/XI	0.0062	0.0333	0.0013	0.0184	-	-	-	-
II/I	0.0708	0.1333	0.0622	0.373	-	-	-	-
III/I	0.1592	0.2333	0.0989	0.4378	-	-	-	-
III/III	0.0937	0.0333	-0.0556	-0.2618	0.026	0.0217	-0.0066	-0.0605
III/VI	-	-	-	-	0.1559	0.0761	-0.077	-0.3354
III/VII	-	-	-	-	0.0052	0.0109	0.0052	0.104
III/IX	-	-	-	-	0.0156	0.0109	-0.0061	-0.0789
III/X	0.0094	0	-0.0089	-0.1108	0.2651	0.4022	0.13	0.5203
IV/I	0.0177	0.0333	-0.0011	-0.0126	-	-	-	-
V/II	0.0021	0.0333	0.0311	0.7167	-	-	-	-
VI/II	0.0021	0.0333	0.0322	0.7423	-	-	-	-
VI/X	0.001	0	-0.0011	-0.0356	0.005	0.0108	0.0052	0.1061
VII/III	0.0104	0.0333	0.0222	0.2691	-	-	-	-
VIII/I	0.1061	0	-0.1	-0.5086	-	-	-	-
VIII/III	0.0624	0.2	0.1333	0.7219	0.0005	0	-0.0007	-0.0323
VIII/X	0.0624	0	-0.0067	-0.0955	0.005	0.0108	0.0051	0.1061
IX/I	0.0177	0.0333	0.0156	0.1769	0.0002	0.0109	0.0106	0.7708
X/III	0.0104	0.0333	0.0222	0.2691	-	-	-	-
XI/VII	-	-	-	-	0.0029	0.0108	0.0077	0.1704
XII/VII	-	-	-	-	0.0353	0.0652	0.0301	0.2043
XII/VIII	-	-	-	-	0.0012	0.0109	0.0096	0.2983
XII/X	-	-	-	-	0.06	0.043	-0.0189	-0.1179
XIII/V	-	-	-	-	0.0005	0.0108	0.0104	0.4842
XV/VI	-	-	-	-	0.0029	0.0108	0.0077	0.1704
XVI/X	-	-	-	-	0.005	0.0108	0.0052	0.1061

Table S5: Tests of neutrality in cultivated and wild flax.

Locus	Population	Tajima’s D	Fu and Li's D2	Fu and Li's F	R2 Test
LuTFL1	All	-0.7254	0.8105	0.23	0.0375*
	Cultivated	-1.4701*	-4.6342**	-3.8800**	0.0401*
	Wild	-0.1403	0.0199	-0.0361	0.1099
LuTFL2	All	2.1806	0.1114	1.0638	0.1319
	Cultivated	1.5637	-0.0399	0.6442	0.1256
	Wild	-1.4443	1.2241	0.4099	0.0708

* rejection of null hypothesis with confidence of 0.05
** rejection of null hypothesis with confidence of 0.01

Table S6: P values of *LuTFL1* I and III positions in the RAD allele frequency spectrum.

Allele	P value relative to diagonal probabilties	P value relative to horizontal probabilties	P value relative to vertical probabilties
I	0.00021091	0.00326645	1.53E-06
III	0.02411791	0.00461858	0.04410392

Table S7: F_{ST} values between northern and southern cultivated flax populations across all and ∂f subsets of RAD loci and enrichment of ancestry information.

	ft	∂f(Nw-Sw) > 0.3	∂f(Sw-Nw) > 0.3	∂f(Nw-Sw) >0.4	∂f(Sw-Nw) > 0.4	∂f(Nw-Sw) >0.5	∂f(Sw-Nw) > 0.5
Mean Fst	0.021229	0.026124288	0.02069484	0.033327468	0.021649736	0.038403004	0.007924316
P value		0.133313331	0.576515303	0.04110411	0.124812481	0.00230023	0.237801693
Ia∂f/Iat		1.208788859	0.987098562	1.18274407	0.384909389	1.300927191	0.308692213

Supplementary Figures

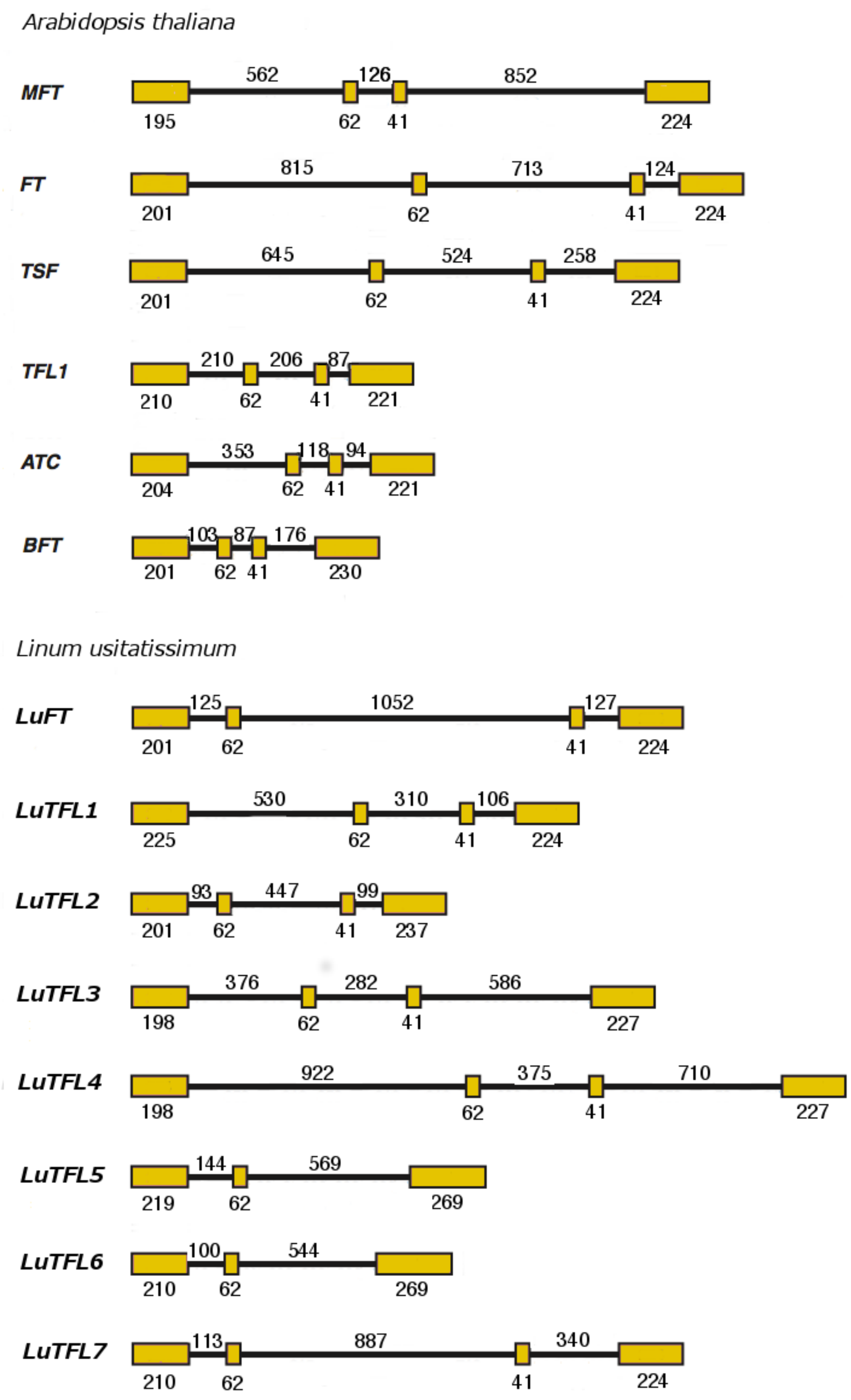

Figure S1: Exon structure of the PEBP family in *Arabidopsis thaliana* and *TFL* homologues in *Linum usitatissimum.*

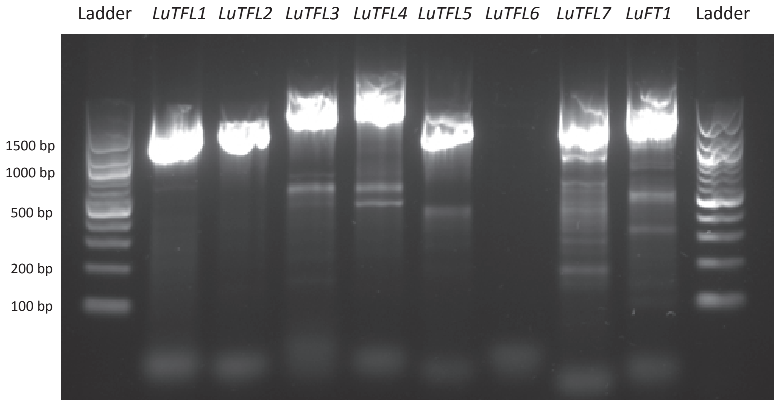

Figure S2: PCR amplicons of putative *TFL1* homolog targets.

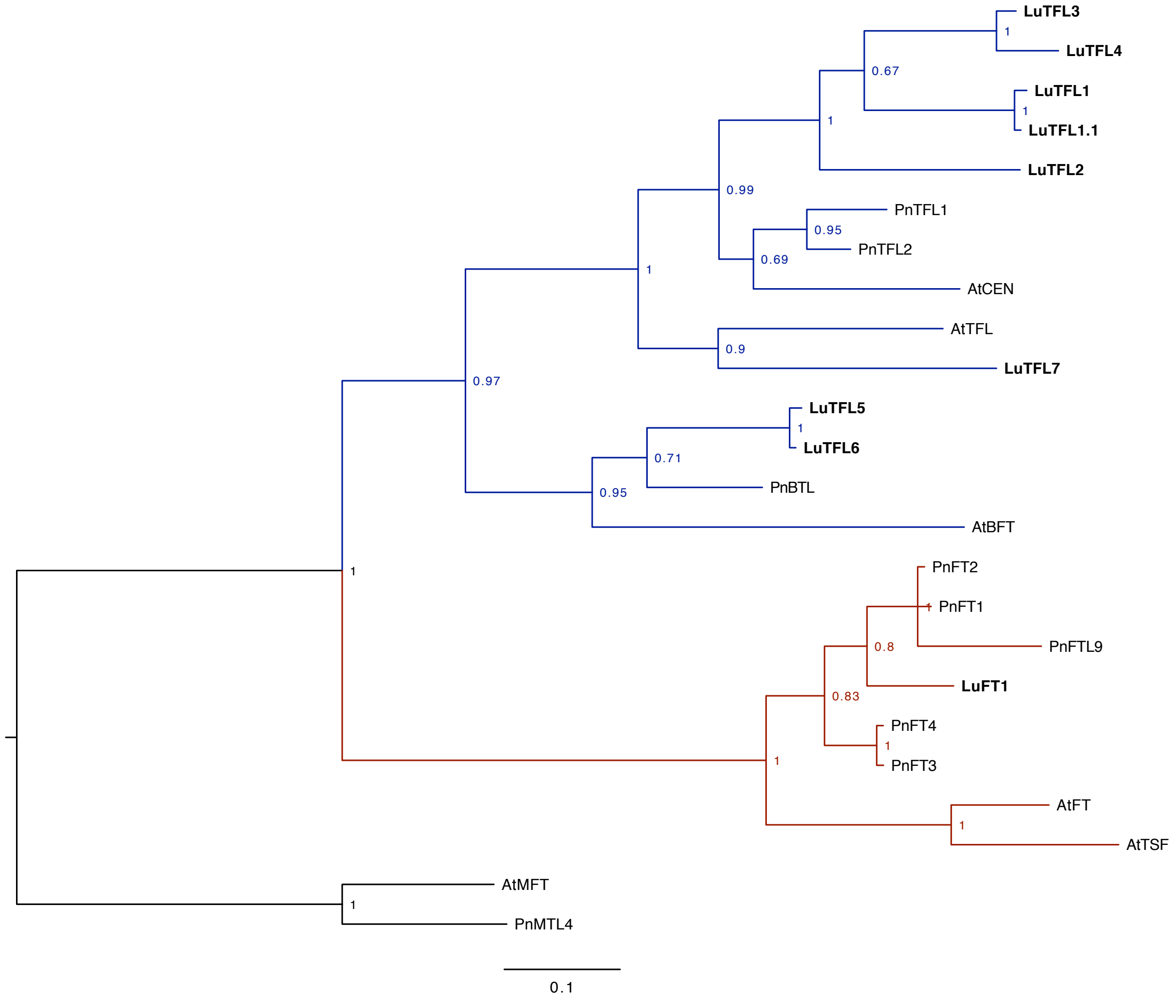

Figure S3: Phylogenetic tree obtained through Bayesian inference represents relationship of PEBP family genes in *Linum usitatissimum*, *Arabidopsis thaliana* and *Populus nigra*. Values by the internal nodes represent clade posterior probability. Gene prefixes indicate the organism in which locus was identified (At – *A. thaliana*, Lu – *L. usitatissimum*, Pn – *P. nigra*).

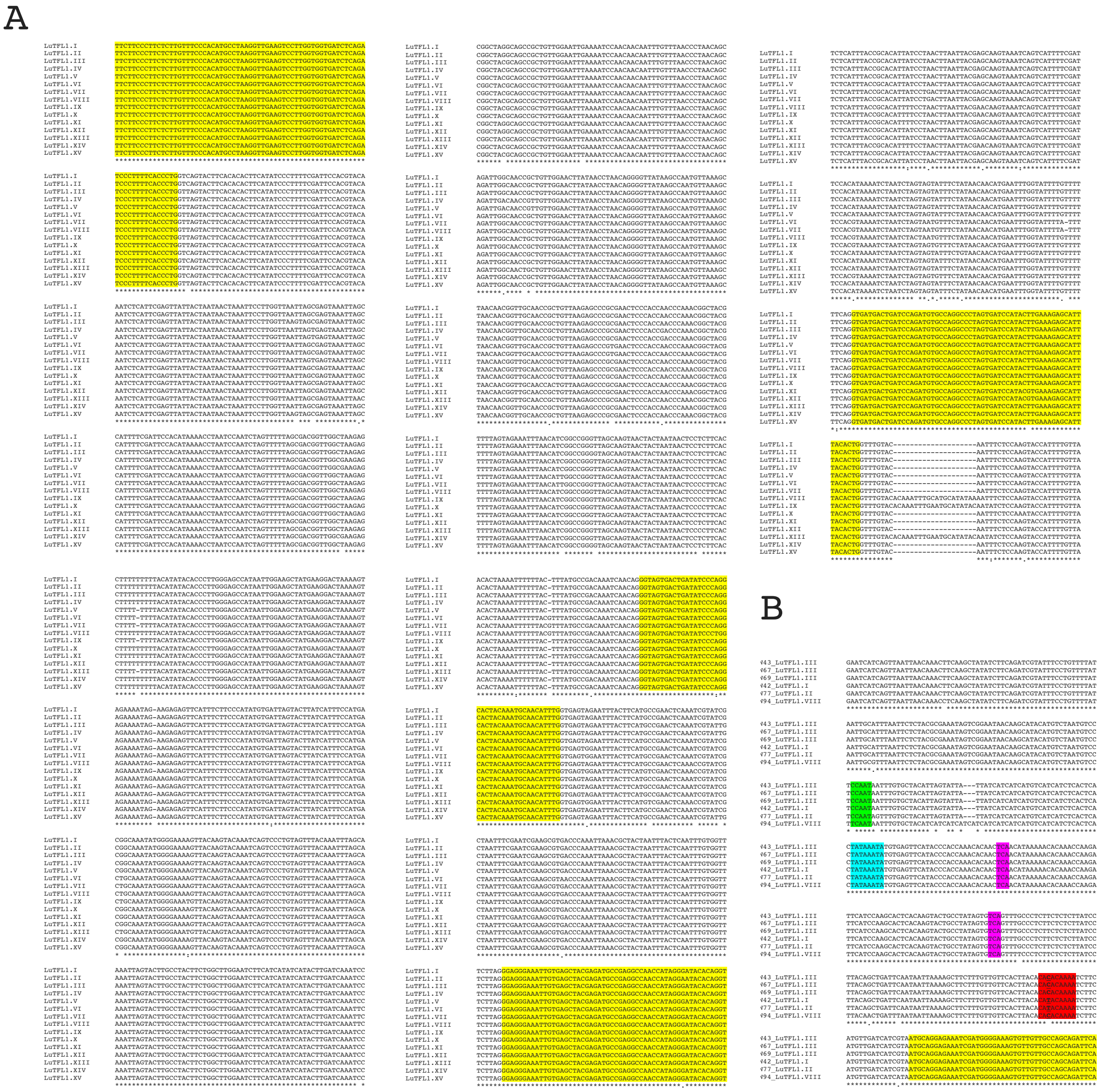

Figure S4: Alignment of *LuTFL1* sequences used in this study. A. Alignment representing 15 different haplotypes of *LuTFL1* gene. Putative exons were marked with yellow highlights. B. Alignment of 5'UTR region of *LuTFL1* gene as extension of four important haplotypes (I, II, III and VIII). Promoter motifs are marked with green, cyan and purple respectively. Transcription factor binding site with putative role in flower development is marked in red.

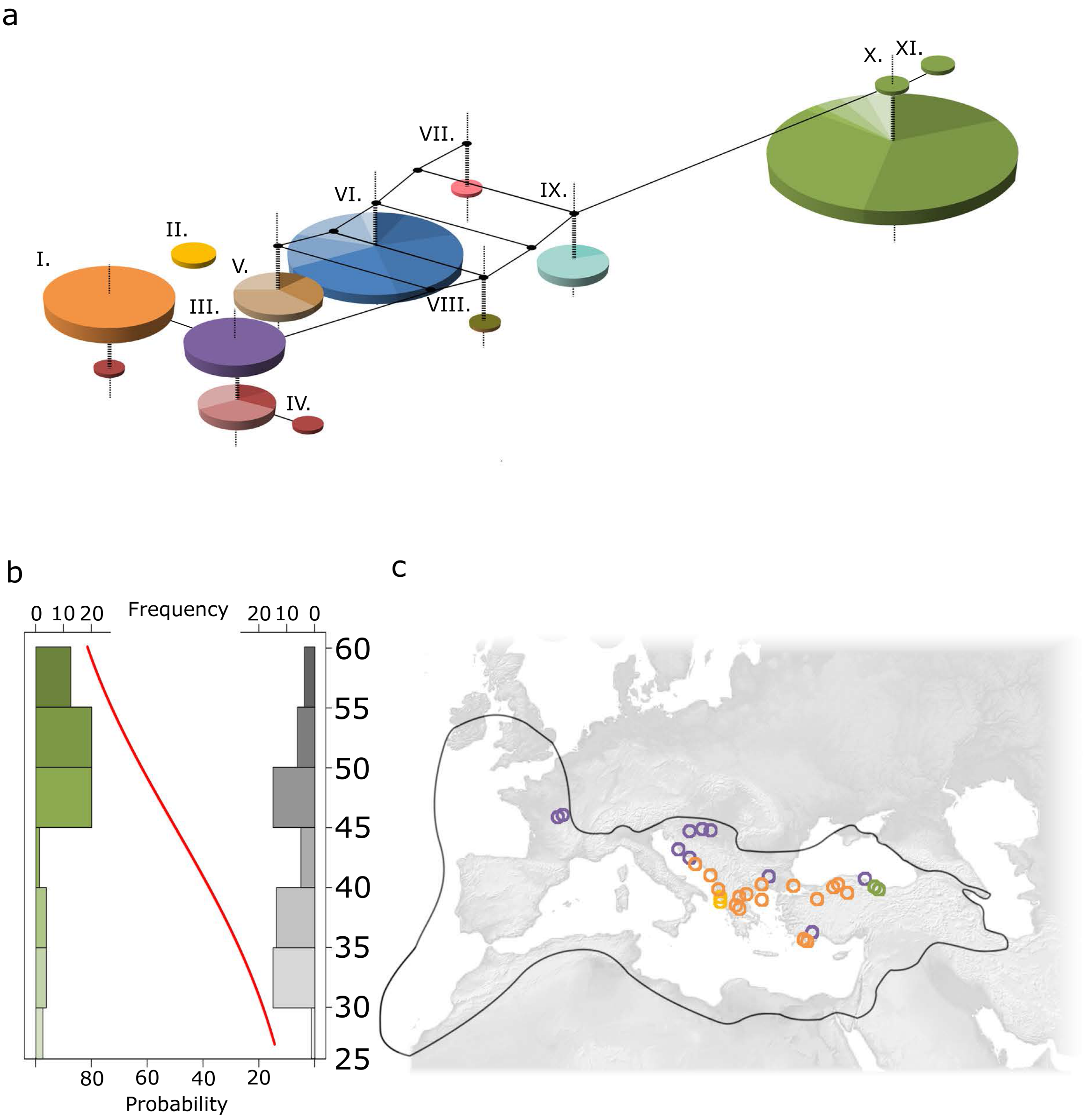

Figure S5: Relationship and geography of cultivated and pale flax according to *LuTFL2* data: A. Splits Tree network of pale (top) and cultivated (bottom) flax, size of nodes is proportional to number of samples with the same haplotype, continuous branches denote molecular distance between, vertical dotted lines link different species within same haplotype. B. Histogram showing latitudinal gradient of *LuTFL2* alleles in cultivated flax, to the left frequency of haplotype XI (green), to the right frequency of all other haplotypes (grey) with fitted logistic regression curve (p-value of 0.000291) reflecting occurrence probability of northern haplotype in latitude gradient. C. Map of Europe marked with wild distribution of pale flax (black line) and pale flax sampling locations (colours correspond to haplotypes in splits network).

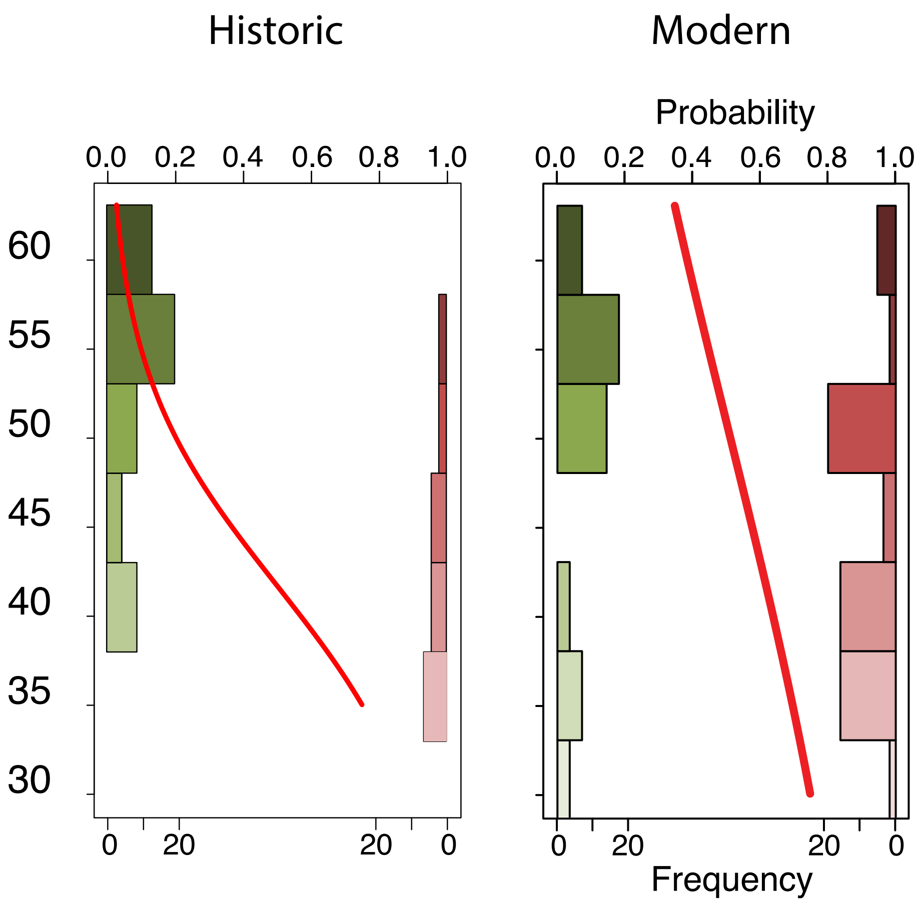

Figure S6: Latitudinal enrichment of *LuTFL1* III in modern and historic flaxes. Histogram showing latitudinal gradient of *LuTFL1* alleles in historic and modern cultivated flax, in green, frequency of northern haplotype cluster (*LuTFL1*.III), in red, frequency of southern haplotype cluster (*LuTFL1*.I) with fitted logistic regression curve (p-value of 0.00144) reflecting occurrence probability of northern haplotype in latitude gradient.

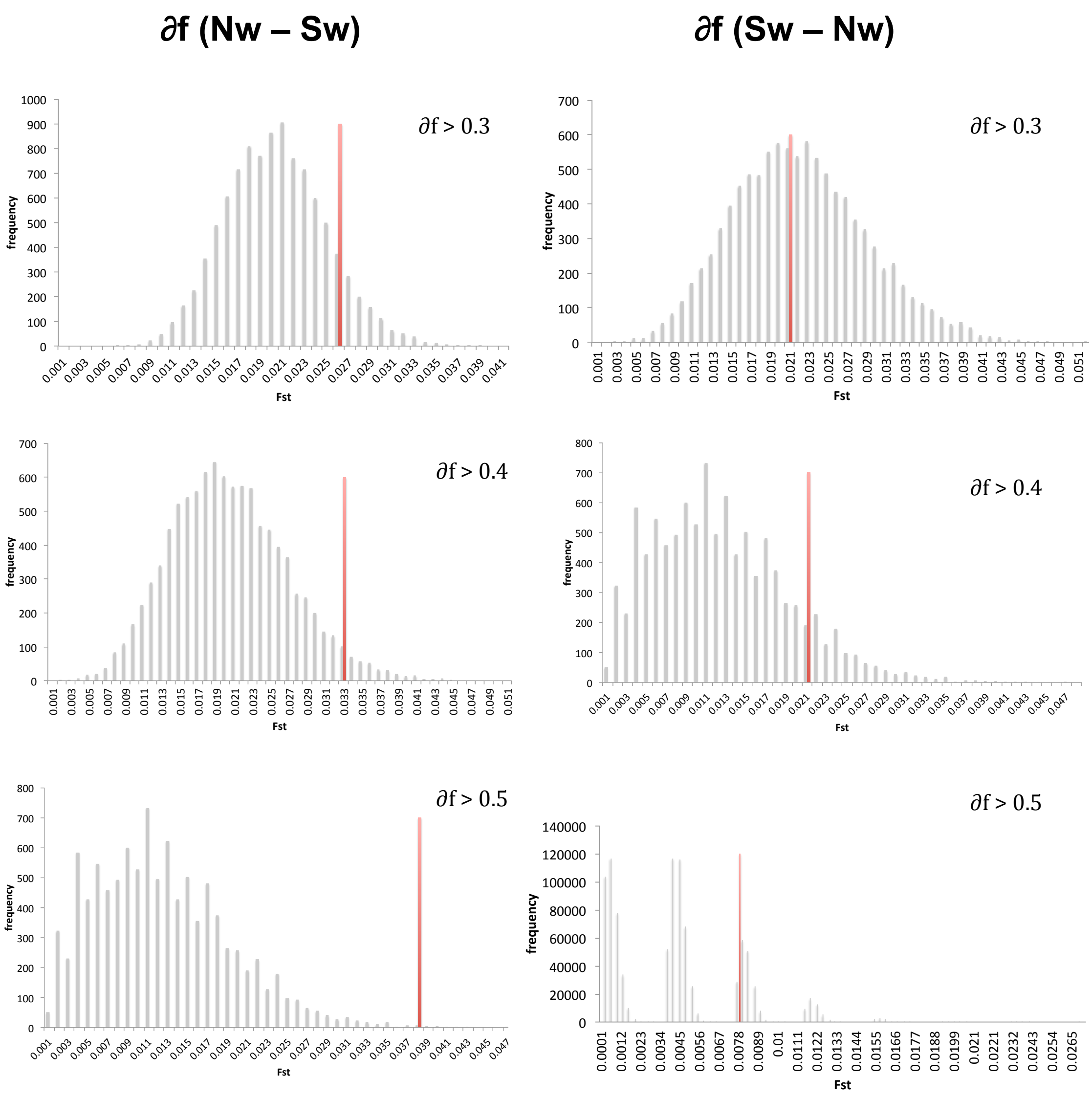

Figure S7: Variant allele test. Average Fst values in cultivated flax of *∂f* loci subsets defined in wild populations shown by red bars. Null distributions shown in grey bars generated by random sampling 10^4 or 10^6 times equal subset sizes from all cultivated flax values. Significance values shown in Table S3.

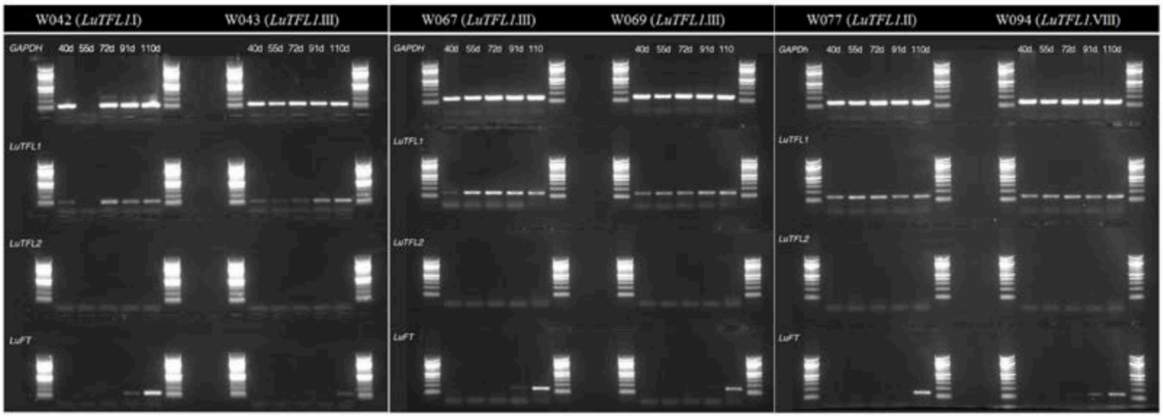

Figure S8: Semi-quantitative RT-PCR expression analysis in leaf and meristem tissue in individuals from six different populations of pale flax. Accession ID and associated *LuTFL1* haplotypes are indicated in the top. Analysis was carried out for *GAPDH* (housekeeping gene as a positive control for constant expression), *LuTFL1*, *LuTFL2* and *LuFT* genes.

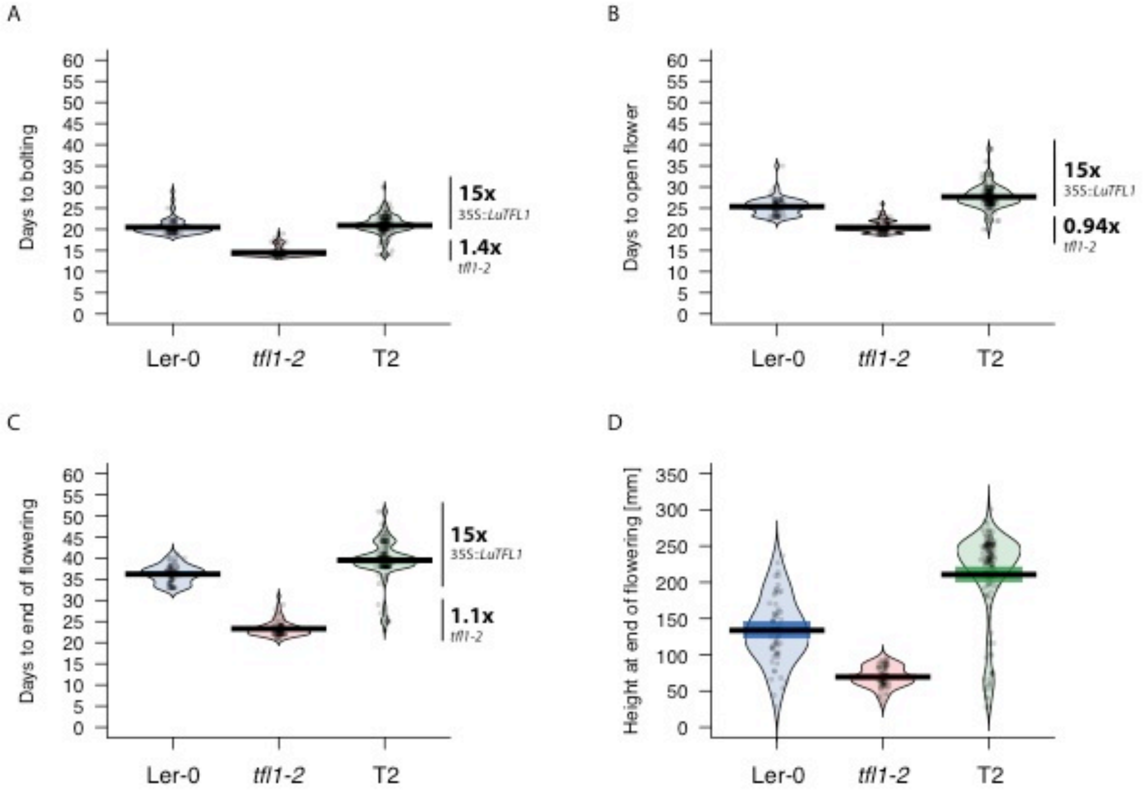

Figure S9: Phenotypic traits of *Arabidopsis thaliana* genotypes *TFL* (Ler-0), no *TFL* (*tfl1-2*) and *LuTFL1 III* (T2). A. Days to bolting. B. Days to flower opening. C. Days to end of flowering. D Height at end of flowering.

Supplementary Methods

Modelling selection over time in *LuTFL1*
Each latitude in Europe is associated with a different time of arrival of agriculture based on archaeological data. The program SELECTION_TIME.pl estimates selection coefficients from dated frequencies (available for download from http://www2.warwick.ac.uk/fac/sci/lifesci/research/archaeobotany/downloads/flax). The model takes as input a series of dated frequencies, mating strategy of the organism, whether the data is phenotype or allele frequency and whether the trait under selection is dominant or recessive. The estimation of allele frequency from phenotype function was not required in this study because the input data was allele frequency. To calculate the inbreeding coefficient associated with the mating strategy the program begins with Hardy Weinberg frequencies based on 50% frequency of alleles in a two-allele system, and then iterates through generations making genotypes based on the mating strategy using equations *2*, *3* and *4*.

$$P' = m(P + 0.25Q) + (1 - m)(P^2 + PQ + 0.25Q^2) \quad (2)$$
$$Q' = m(0.5Q) + (1 - m)(2PR + 0.5Q^2 + PQ + QR) \quad (3)$$
$$R' = m(R + 0.25Q) + (1 - m)(R^2 + QR + 0.25Q^2) \quad (4)$$

Where P, Q and R denote the proportions of the dominant homozygote, heterozygote and recessive homozygote individuals in a population respectively, m denotes the mating strategy defined as the probability of a selfing event, and P', Q' and R' denote the proportions of P, Q and R in the next generation. This iteration causes genotype frequencies to equilibrate to their expected frequencies under the mating strategy m. Each generation the inbreeding coefficient F is calculated as determined by the under-representaton of heterozygotes and over-representation of homozygotes in the case of inbreeders following equation *5*.

$$F = \frac{R - q^2}{pq} \quad (5)$$

Where p and q are the proportions of the dominant and recessive alleles respectively. The iterations continue until consecutive values for F differ by less than 1 x 10^{-10}, and the frequency equilibrium of genotypes has been reached.

The program then takes a pair of input observed dated allele frequencies as start and stop frequencies. The inbreeding coefficient F is used to determine the initial genotype frequencies using the equations *6, 7* and *8*.

$$P = p^2 + Fpq \quad (6)$$
$$Q = 2pq(1 - F) \quad (7)$$
$$R = q^2 + Fpq \quad (8)$$

Beginning with the initial (lower frequency), a number of generations equal to the date difference are iterated through using equations *9, 10* and *11.*

$$P' = (1 - k)m(P + 0.25Q) + (1 - k)(1 - m)(P^2 + PQ + 0.25Q^2) \quad (9)$$
$$Q' = (1 - k)m(0.5Q) + (1 - k)(1 - m)(2PR + 0.5Q^2 + PQ + QR) \quad (10)$$
$$R' = m(R + 0.25Q) + (1 - m)(R^2 + QR + 0.25Q^2) \quad (11)$$

Where k is the selection differential taking values between -0.000001 and -0.999999, and selection is of the dominant allele p in this case to reflect the existence of the transcription-

factor binding site in the 5' upstream region of the *LuTFL1* III allele under selection. The selection coefficient s is then described by equation *12*.

$$s = 1 - \frac{1}{(1-k)} \tag{12}$$

The variable k_i is designated as either k_u or k_o depending on whether it's implementation in equations *9* and *10* lead to a final gene frequency of p (p_f) that is under or over the real observed final gene frequency (p_{fo}). The initial values of k_u and k_o are -0.000001 and -0.99999 respectively. Iterative values of k are calculated using equation *13*.

$$k_i = k_u + \frac{k_o - k_u}{2} \tag{13}$$

$$k_{u+1} = k_i \ for\, p_{fi} < \ p_{fo} \tag{14}$$

$$k_{o+1} = k_i \ for\, p_{fi} > \ p_{fo} \tag{15}$$

The iterations continue until consecutive estimates of k_u and k_o differ by less than 1x 10^{-6}. Estimates of k were made for all pairs of dated frequencies.

To account for sample error, we generated upper and lower bounds of s for dated frequencies. To do this we used a Beta distribution to estimate the 95% range of possible underlying frequencies that could have given rise to the observed number of genotypes in our samples assuming a binomial process of x observations in n samples, where x equates to the number of samples carrying the *LuTFL1* III allele, and n equates to the total number of samples at a specific latitude. We then calculated all possible values of s using the upper and lower sample number bounds respectively.

Post-domestication gene flow modelling

We utilized the ancestral information content concept to employ a high variant test to explore possible gene movement between populations when there may be complex confounding movements masking a general signal that might be detected through f4 statistics. Our starting premise is that loci that have become highly differentiated between potential source populations through drift may be exploited as markers for that population to probe gene movements between specific populations in the face of multiple general movements. This concept has recently been used to investigate maize genome ancestry, and generally to identify ancestry informative markers. The probability that a certain allele will be involved in a gene flow process is expected to be proportional to its frequency in the population, and the likelihood such alleles would result in a detectable impact in the recipient population would be expected to increase as the difference in frequencies (∂f) between the population increases. In a case in which two sets of two populations have differentiated from each other independently, we would expect highly differentiated loci in one population pair to be independent to the extent of differentiation of the corresponding loci in the other population. Using those terms, we assume the relationship of branch lengths to be as follows:

$$i + j \approx w + x$$

$$k + l < y + z$$

We describe the subset of differentiated loci as ∂f where the delta term refers to the minimum extent of difference between frequencies in one population pair, and f_t the differences across all loci. However, instance in which the following relationship is satisfied:

$$i + j < w + x$$

would suggest a non-independent process of differentiation between the sets of populations. By selecting subsets of loci that are specifically high in one population (e.g. population C in figure S4), we can explore the independence of differentiation with specific respect to that donor population.

The expected relationship between our flax populations is that firstly wild southern and northern populations diverged, subsequently flax became domesticated in the south, and then spread to the north diverging from its respective southern population independently from the wild diversification. It is reasonable to expect that the southern cultivated population should resemble the southern wild population, but that should not matter for this analysis. However, we first examined the differentiation of all loci through Fst values between the respective northern and southern populations of wild and cultivated flax to check for broad correlation of frequencies that would compromise the underlying assumption of independent differentiation and possibly indicate a hitherto undetected signature of structure. The Fst values between the wild populations and the cultivated populations appear to be uncorrelated (r = 0.098), supporting the notion that differentiation has been largely independent in wild and cultivated flaxes. We then identified three subsets of loci in which the difference in frequency (∂f) between northern and southern wild populations was more than 0.5, 0.4 or 0.3, with the higher allele in the northern population. This process was repeated in the wild southern population. The average Fst value across those loci subsets was then calculated for the cultivated population. To compare the ∂f subset mean Fst obtained in cultivated flax populations with the total null background of markers we sampled 10000 times random subsets of the same sample size from all Fst values between cultivated populations and calculated means producing null mean Fst distributions. In the case of ∂f >0.5 and higher frequency in southern wild populations, we resampled 1000000 times because of the low number of loci recovered that discriminate southern populations to this level. We found that loci with alleles that are of discriminatingly high frequency in southern wild populations were agnostic in cultivated populations. However, in the case of northern wild populations we find a strongly significant relationship between alleles that discriminate wild populations and those that discriminate cultivated populations, suggesting genetic contact between the northern cultivated and wild populations. We examined the ancestry information content of the ∂f loci subsets using the I_a ($I_{a\partial f}$) statistic relative to the information content of all loci (I_{at}). These results echoed our variant allele test, with increasing high values of ∂f being associated with an increased ancestral information content of the northern but decreasing in the southern wild population. This results are congruent with a process in which cultivated populations passed through the southern wild flax range in which higher frequency alleles were more more likely to become incorporated in both the resultant southern and northern cultivated flax populations and hence be poor ancestral discriminators. The high ancestral information content of the discriminatingly higher northern wild populations is congruent with some distinct ancestral contribution to northern cultivated flaxes. Together these data suggest a process of gene movement from the northern wild flaxes into northern cultivated populations.

Simulating flax growth with different *LuTFL1* expression levels

The *PGROWTH* model simulates flax growth and development (http://www2.warwick.ac.uk/fac/sci/lifesci/research/archaeobotany/downloads/flax). The key event in the model determining plant architecture is flowering time. A change in the flowering time impacts on plant stature and branching pattern. A script in the *R* programming language called *PGROWTH* has been written by RG to simulate the impact of the photoperiod-dependent pathway on flax

architecture during its development. It is an implementation of observations made by McGarry and Ayres on the impact of *FT*/*TFL* expression on tomato plant architecture, and represents a simpler system than that of Prusinkiewicz In order to simulate flowering on second order branches we used a three-threshold approach, the first to initiate branching, the second to initiate flowering in lateral branches and the third to terminate flowering. This contrasts with Prusinkiewicz who used a system of reversions between meristem states to achieve a wider range of architectures than occurs in our model, or with flax. The thresholds we used were trained to recapitulate type of growth and inflorescence architecture characteristic for flax. The molecular basis of the model assumes that *TFL* and *FT* compete for the same binding site, and that when *FT* exceeds *TFL* expression floral initiation will occur. The model takes as input variables, the germination date, the season length, the minimum daylength required to express *FT,* the level of TFL expressiom, and the geographical latitude of the plant simulated. The model components were as follows:

- *Space*: Latitude is an input variable to the model in order for daylength to be calculated. Latitude is input as the variable α in radians as described in equation (*16*)

$$\alpha = \frac{lat}{57.296^{\circ}} \qquad (16)$$

- *Time*: The model increments on a daily basis, with the first day equating to the 1st March in this study. The 'date' is converted to the variable β in radians using equation *17.*

$$\beta = \frac{2\pi(x+59)}{356} \qquad (17)$$

- *Daylength*: This is calculated using an empirical formula[74] based on the date in radians (β), and input latitude in radians (α):

$$tan(\alpha) * tan\left(-\frac{\pi(22.9133*cos(\beta)+4.02543*sin(\beta)-0.3872*cos(2*\beta)+0.052*sin(2*\beta))}{180}\right) * \frac{180}{\pi} \qquad (18)$$

- *Vegetative growth*: During this phase of simulated plant growth stems and adjacent leaves grow indeterminately and uniformly. In this study scenario, plants grew 1 cm per day and leaves were produced every two days.
- *Transition*: The vegetative phase ends when the expression of *FT* is greater than the expression of *TFL* triggering the transition from vegetative to generative growth.
- *TFL and FT expression*: *TFL* is assumed to be constantly expressed in the model at a set number of units per day, while *FT* is expressed only when the user-defined daylength threshold is met. *TFL* expression levels take a value between 0-100 units in this model. *FT* expression is a linear function of the number of leaves below each meristem, each leaf contributing a single unit, therefore increasing expression levels as vegetative growth progresses. Note that it is a simplification in this model that *FT* producing leaves are insensitive to increasing daylength in regard to the quantity of *FT* produced. Given the close recapitulation of real flax architectures produced by the model, this appears to be a reasonable simplification.
- *Generative growth*: During the generative phase the rates of stem growth remains the same as with the vegetative phase, but instead of producing leaves every second day the main stem produces leafy-bracts and axillary stems (floral branches) alternately. Axillary branches grow in a vegetative manner until a second threshold is reached, which was set to when *FT* expression is 1.1 times higher than the *TFL*. Once this second threshold is reached on the axillary stems leafy-bracts and pedicels are generated alternately every 2 days. The generative growth phase continues producing

axillary branches from the main stem and pedicels on the axillary stems until the third termination threshold has been reached, which is when *FT* is 1.4 times *TFL*. Alternatively, all meristems are terminated with an inflorescence after a defined period of time, in this analysis the season length was set to 150 days. The combination of thresholds used in this model were selected to produce a racemose corymb that closely reflects flax inflorescence.

The model executes beginning at day 1, which corresponds to 1st March. The day length of each day is calculated using equation (18) based on latitude and date as defined by the day number. At a user-defined date a simulated germination occurs which is defined as the initiation of vegetative growth in this model. During this phase each day the plant accrues 1cm length along a single un-branched main stem axis, and every other day a leaf is produced. In this model *TFL* is user-defined to a value between 0-100 units and produced from the first day of vegetative growth at a constant level. The expression level of *TFL* sets the threshold that *FT* expression must reach to trigger generative stage. When a threshold daylength is reached, defined by 14 hours in this study, the vegetative plant begins to express *FT*. The quantity of *FT* is dependent on the number of leaves in the plant, with each leaf producing one unit of *FT* daily. The vegetative phase of growth continues until the leaf production of *FT* is greater than *TFL* expression, upon which point the generative mode of growth is assumed. In the generative phase the simulated plants generate axillary branches on the main stem. Axillary stems after reaching 1.1 *FT*:*TFL* ratio switch to generative phase in which flowers are produced. In the generative phase the simulated plant on the main stem produces leafy-bracts and axillary stems (floral branches) alternately every fifth day. On the axillary stems leafy-bracts and pedicels are generated alternately every 5 days. In this simulation growth was terminated with a terminal inflorescence either by end of season defined as 150 days after the germination date (user-defined date) or by the ratio of *FT*:*TFL* reaching 1.4.

The script includes graphical functions to visualize the output architecture. In this study the following parameters were explored: latitudes N35° and N60°, *FT* expression threshold set to 14 hours and *TFL* expression was set from 0 to 100 which should cover all possible architectures achievable under the combination of thresholds used in this study.

Different input settings yielded different inflorescence architecture. These architectures reflected closely sketches of different flax convarieties made by Kulpa and Danert. Generally, the effect of increased latitude was to produce shorter bushier plants for a given *FT/TFL* floral initiation threshold, while an increase in the threshold value gave rise to taller, less branched plants at any one latitude. This finding is consistent with the notion that an increased expression of *TFL* or reduced expression of *FT* would likely give rise to tall phenotypes more suitable for fibre use. However, the model also demonstrated the expectation that a larger range of architectures would be expected to be viable at higher latitudes because at low floral initiation ratios short plants would flower earlier in the year, and at high initiation ratios tall phenotypes would be able to flower later in the year. Therefore given a selection pressure to adapt to latitude by strengthening the signal to delay flowering in order to achieve a similar date of flowering, we would expect taller phenotypes to prevail.
